# Synthesis of salcaprozate sodium and its significance in enhancing pancreatic kininogenase absorption performance

**DOI:** 10.1002/prp2.1186

**Published:** 2024-03-21

**Authors:** Zhong Lv, Qian‐Dong Luo, Zhang‐Yong Tang, Xiao‐Hu Lv, Tao Wu, Ling‐Kai Huang, Can Tang

**Affiliations:** ^1^ School of Pharmacy Chengdu Medical College Chengdu China; ^2^ Department of Research Chengdu Pu Kang Wei Xin Biotechnology Co., Ltd Chengdu China; ^3^ Chengdu Pu Kang Wei Xin Biotechnology Co., Ltd Chengdu China; ^4^ Department of Research Debor (Chengdu) Biotechnology Co., Ltd Chengdu China; ^5^ Department of Quality Chengdu Pu Kang Wei Xin Biotechnology Co., Ltd Chengdu China; ^6^ School of Science and Technology Chengdu Medical College Chengdu China

**Keywords:** Caco‐2 cells, enhancing absorption, enzyme‐linked immunosorbent assay, pancreatic kininogenase, pharmacokinetics, Salcaprozate sodium

## Abstract

We conducted pharmacokinetic research wherein salcaprozate sodium (SNAC) was utilized as a penetration enhancer by incorporating it into pancreatic kininogenase (PK) to improve the bioavailability of pancreatic kininogenase enteric‐coated tablets. We conducted in vitro studies on PK using the Caco‐2 cell model and quantified PK levels using the enzyme‐linked immunosorbent assay (ELISA) method. We conducted methodological verification by blending SNAC and PK powders into enteric‐coated capsules, and studied the pharmacokinetic characteristics. Based on the PK transport assay, the cumulative permeation rates of the test group that employed a SNAC to PK ratio of 32:1, 16:1, 8:1, 4:1, and 2:1 were 13.574%, 7.597%, 10.653%, 3.755%, and 2.523%, respectively. We conducted a uniformity test on the powder that contained a blend of SNAC and PK. The relative standard deviations (RSDs) for both the power containing SNAC and the power not containing SNAC were less than 10%. Based on the methodological verification, in vivo pharmacokinetic study of PK met the experimental requirements. As indicated by the results of in vivo pharmacokinetic research on rats, the test group (This group used SNAC) had a PK AUC_0–12 h_ of 5679.747 ng/L*h and *t*_1/2_ of 4.569 h, while the control group (This group did not use SNAC) had a PK AUC_0–12 h_ of 4639.665 ng/L*h and *t*_1/2_ of 3.13 h. This study has established a low‐cost, environmentally friendly, and safe SNAC synthesis route with high process yield suitable for industrial production. SNAC demonstrates an absorption‐enhancing effect on PK, and the optimal ratio of SNAC to PK is determined to be 32:1.

## Graphical abstract

Research flow chart.

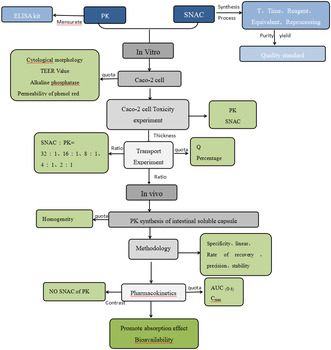

**Abbreviations**
ALP: alkaline phosphatase
AP: apical
BL: basolateral
Caco‐2: Cell the human colon adenocarcinoma cell line
CDI: carbonyldiimidazole
DCM: dichloromethane
DMF: dimethylformamide
DN: diabetes nephropathy
EA: ethyl acetate
Et_3_N: Triethylamine
GLP‐1: glucagon‐like peptide‐1
MeOH: methanol
OD: optical density
Papp: apparent permeability coefficient
PE: petroleum ether
PK: pancreatic kininogenase
TEER: trans‐epithelial electrical resistance
THF: tetrahydrofuran

## 1 INTRODUCTION

Diabetic nephropathy (DN) is one of the most common microvascular complications of diabetes mellitus. Its clinical manifestations vary according to the stages of the disease. It is mainly characterized by varying degrees of proteinuria and a decline in kidney function. The incidence of early‐stage diabetic nephropathy is approximately 30%^1^; hence, necessary interventions during this stage can prevent or delay the progression of DN.^2^ PK has shown significant efficacy in the early treatment of diabetic nephropathy. It demonstrates the capability to eliminate or markedly reduce urinary protein levels, effectively decelerating the progression of diabetic nephropathy and preserving kidney function. However, its primary shortcomings in comparison to other drugs presently available in the market encompass low PK bioavailability and poor patient compliance, necessitating additional research.

PK belongs to the class of proteolytic enzymes and plays a significantly important role in the human kinin system. Pancreatic kininases can degrade into kinins, which are highly potent vasoactive substance capable of dilating renal blood vessels. They can promote the excretion of water and sodium, lower blood pressure, and increase renal blood flow. Additionally, they reduce pressure in the renal efferent arterioles and capillary resistance, decrease the quantity of protein excreted in urine, and improve kidney function.^3^, ^4^, ^5^ Presently, the market offers enteric‐coated PK tablets and injectable PK formulations. The bioavailability of enteric‐coated PK tablets remains notably limited, posing a cost escalation concern for pharmaceutical enterprises. This limitation may necessitate consumers to ingest higher individual doses or even undergo multiple administrations for efficacy. Injectable PK, on the other hand, faces issues related to poor patient compliance. The objective of this study was to investigate the absorption‐enhancing effect of the permeability enhancer—SNAC—on PK absorption using the Caco‐2 cell absorption model. Subsequently, SNAC‐containing enteric‐coated PK capsules was formulated and studied for their pharmacokinetic characteristics in rat models. We hope our study can enhance the bioavailability of PK, improve drug stability, and provide essential theoretical foundations for the development of novel oral second‐class PK medications.

## 2 MATERIALS AND METHODS

### 2.1 Material

#### 2.1.1 Instruments

The quality of SNAC was tested using a high‐performance liquid chromatography system (Agilent Technologies [China] Co., Ltd. DEACX12727). Cell morphology was observed using an inverted phase contrast microscope (Nikon Corporation. TA100‐452331). Microplate reader (Molecular Devices Shanghai Co., Ltd. 2016001847), detects the content of PK utilizing a battery resistance meter (Millipore Sigma. ERS‐2‐01) to measure the cell resistance value. The isolation of CaCo‐2 cells was performed by a low‐speed centrifuge (ANHUI USTC Zonkia Scientific Instrument Co., Ltd. 201500013).

#### 2.1.2 Medicines and reagents

Salicylamide (Huayin Jinqiancheng Pharmaceutical Co., Ltd., Y366220101) is a synthetic raw material for SNAC. PK (Sichuan Deebiotech Co., Ltd. D08‐220501. 90 U/mg) is the research object.. DMEM medium (Chengdu Yufeng Boyan Biotechnology Co., Ltd.06‐1055‐57‐1ACS) is the culture medium for CaCo‐2 cells. Enhanced CCK‐8 (Beyotime Biotechnology Co., Ltd., Shanghai City. C00423) is used for cytotoxicity experiments. Phosphate‐buffered saline (PBS) solution (Chengdu Yufeng Boyan Biotechnology Co., Ltd. 02–024‐1ACS.) is the diluent. Film coating powder (Chengdu Taishan Pharmaceutical Co., Ltd. 20221201) is used for PK enteric‐coated capsule coating.

#### 2.1.3 Cell strains

Caco‐2 human rectal adenocarcinoma cells (Meiwan Biotechnology Co., Ltd., Shanghai City) were used for PK in vitro study.

#### 2.1.4 Animals

Eighteen male Sprague–Dawley (SD) rats weighing 200 ± 10 g each were provided by Chengdu Dossy Experimental Animals Co., Ltd. (SCXK 2020‐030).

### 2.2 The synthesis of SNAC

#### 2.2.1 Information of SNAC

SNAC, also known as salcaprozate sodium, has a molecular formula of C_15_H_20_NNaO_4_, a molecular weight of 301.31, and a Chemical Abstracts Service (CAS) registry number of 203787‐91‐1.

#### 2.2.2 The synthesis route of SNAC is shown in Figure 1

**FIGURE 1:**
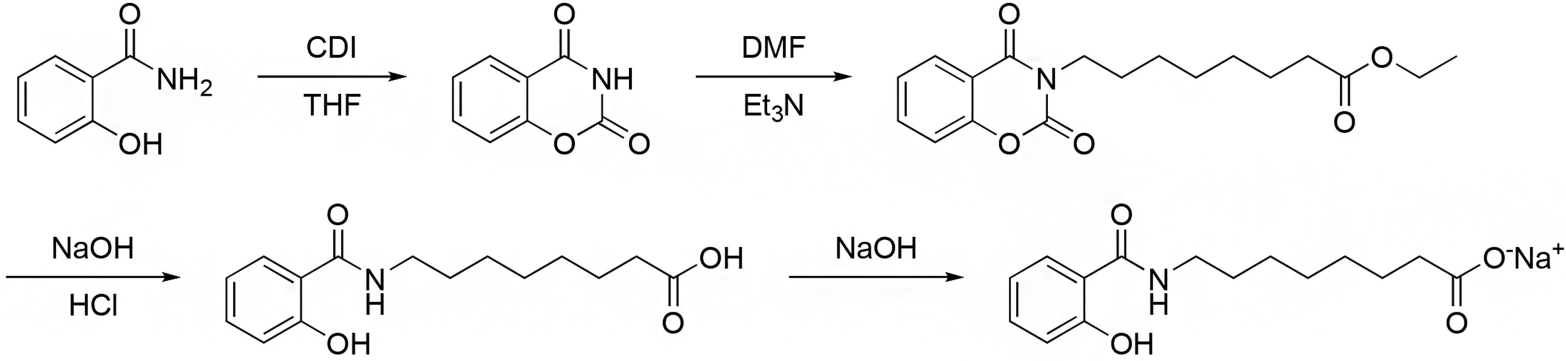
The synthetic scheme of SNAC.

Using salicylamide as the starting raw material, intermediate 1,3‐benzoxazine‐2,4(3H)‐dione was prepared. Subsequently, triethylamine provided alkalinity, and a substitution reaction occurred between 8‐bromooctanoic acid ethyl ester and intermediate 1, yielding intermediate 2. Next, under alkaline conditions, intermediate 2 underwent hydrolysis and ring‐opening reactions, with pH adjustment using hydrochloric acid, resulting in intermediate 8‐(2‐hydroxybenzamido)octanoic acid. Finally, sodium hydroxide provided alkalinity, yielding the desired product, sodium 8‐(2‐hydroxybenzamido)octanoate (commonly known as salcaprozate sodium [SNAC]).

#### 2.2.3 The synthesis of intermediate 1,3‐benzoxazine‐2,4(3H)‐dione

Sixty milliliters of THF was measured, put into a round‐bottom flask, stirred, and added with 10 g of salicylamide and 14.2 g of carbonyldiimidazole. The mixture was kept overnight at 25°C, monitored by a thin layer chromatography (TLC) plate (PE: EA = 1:1). Upon completion of the reaction, an equal volume of water was added, resulting in solid precipitation. The solid was then filtered, dried, yielding a 93.5% recovery rate.

#### 2.2.4 The synthesis of intermediate 2

Five grams of the intermediate 1,3‐benzoxazine‐2,4(3H)‐dione were dissolved in a volume of dimethylformamide (DMF) equal to five times its quantity, placed into a round‐bottom flask, slowly stirred, and added with 7.8 g of triethylamine and 9.2 g of 8‐bromooctanoic acid ethyl ester. The mixture was reacted at 55°C–65°C for 6–10 h, then cooled to room temperature. Hydrochloric acid at a concentration of 1 mol/L was added into the reacted mixture to precipitate the solid. Filtration by suction was performed and the filter cake was rinsed with water. Subsequently, the filter cake was pulped twice using a mixture of methyl tert‐butyl ether and n‐heptane in a 5:1 ratio and filtered by suction. Next, the filter cake was rinsed with n‐heptane. The solid was dried at 55°C for approximately 4 h. The yield was 94.9%.

#### 2.2.5 The synthesis of the intermediate 8‐(2‐hydroxybenzamido)octanoic acid

A 4.3 g quantity of sodium hydroxide was dissolved in 10 times its weight of water, stirred, and then combined with 8 g of intermediate 2. The mixture was reacted at 90°C–100°C for 3–6 h, monitored using the TLC plate (DCM: MeOH = 10:1). Upon completion of the reaction, the mixture was subsequently cooled to room temperature, following which impurities were extracted once using dichloromethane (DCM). Verification was performed via TLC analysis. The aqueous phase was then adjusted to a pH range of 3–5 using 3 N hydrochloric acid, resulting in the precipitation of solid material. After filtration by suction, the solid was rinsed with water and dried at 60°C for approximately 4 h, yielding a 94.0% yield.

#### 2.2.6 The synthesis of sodium 8‐(2‐hydroxybenzamido)octanoate (SNAC)

The intermediate 8‐(2‐hydroxybenzamido)octanoic acid, weighing 5 g, was placed into a small amount of isopropanol and stirred, and then a sodium hydroxide solution of 0.7 g was added dropwise. Subsequently, the remaining isopropanol (totaling 8 V) was also added. The mixture was slowly cooled with stirring to induce crystallization, and then filtered under suction. The resulting filter cake was rinsed with isopropanol and subsequently dried at 60°C for 2–4 h, yielding a 90.8% yield.

### 2.3 The effect of SNAC on the intestinal absorption of PK

Caco‐2 cells originate from cell lines of human colonic and rectal cancer.^6^ Their various physiological and tissue morphological characteristics, as well as the secretion of related functional enzymes, are similar to the epithelial cells of the normal human small intestine mucosa. Additionally, they possess similar enzyme systems related to the epithelial cells of the human small intestine mucosa.^7^ Over the past 30 years of development, as a model for in vitro cultivation of intestinal mucosal epithelial cells, Caco‐2 cells have been widely used in research on in vitro drug studies, such as investigations into intestinal absorption, distribution, metabolic functions, and in vitro excretion functions.^8^, ^9^, ^10^ Therefore, the Caco‐2 cell model is suitable for studying the cellular absorption and uptake of drugs intended to be absorbed in the intestine, as well as specific transmembrane transport processes.

#### 2.3.1 Cytotoxicity experiment using CCK‐8

Logarithmic phase Caco‐2 cells were incubated in 96‐well plates at a concentration of 5 × 10^3^ cells per well and placed in a 37°C, 5% CO_2_ incubator for cultivation. After 24 h of cultivation, cells were rinsed with PBS. For SNAC cytotoxicity experiment group, 90 μL of fresh culture medium and 10 μL of SNAC solution at concentrations of 14, 12, 10, 8, 6, 4, and 2 mg/mL were added. For the PK cytotoxicity experiment group, 90 μL of fresh culture medium and 10 μL of PK solution at concentrations of 18, 16, 12, 10, 8, 6, 4, and 2 mg/mL were added. Three replicate wells were prepared with corresponding concentrations for the measurement, and the culture lasted for 6 h. After the incubation, the cellular waste liquid was removed by suction, and 90 μL of fresh culture medium and 10 μL of CCK8 reagent were added. The mixture was incubated in a 37°C, 5% CO_2_ incubator for 1 h. The absorbance was measured using the microplate reader at a wavelength of 450 nm. The experiment consisted of two groups: a blank control group without cells, and a negative control group with cells but no drug solution. Cell viability was calculated based on the measured absorbance using the following formula:

(1) $$\text{Cell survival rate}\;\left(\%\right)=\frac{\mathrm{As}-\mathrm{Ab}}{\mathrm{Ac}-Ab}\times 100\%$$

In this formula, *As* represents the absorbance value of the experimental wells, *Ac* represents the absorbance value of the control wells, and *Ab* represents the absorbance value of the blank wells.

#### 2.3.2 The establishment of the Caco‐2 cell model

Caco‐2 cells were incubated in the Transwell inserts and placed on a 24‐well plate for culturation and growth. The AP inner surface of the Transwell inserts contained approximately 0.5 mL of culture medium, while the BL side contained approximately 1.5 mL of culture medium. The cells were then cultured for 21 days. The Caco‐2 cell model followed the model established by Lv Xiaojun et al.^11^

#### 2.3.3 Measurement of Caco‐2 Cell monolayer TEER

First, the Millicell‐ERS electrical resistance meter was used to calibrate the resistance and voltage. Initial measurements involved the blank Transwell wells without Caco‐2 cells, followed by determining the transmembrane resistance values of the Transwell wells with cultured Caco‐2 cells. The actual resistance value of the Caco‐2 cell monolayer was calculated using the following formula.

(2) $$\text{TEER}=\left(R-\mathrm{Rb}\right)\times A\;\left(\Omega \cdot {\mathrm{cm}}^{2}\right)$$

Among them, *R* represents the actual resistance value, *Rb* is the value of the blank Transwell wells, and *A* is the membrane area of the polycarbonate film (0.33 cm^2^).

The resistance values were measured on the 2nd, 5th, 7th, 10th, 15th, 21st, and 22nd days after seeding Caco‐2 cells into Transwell inserts.

#### 2.3.4 Determination of ALP activity

On the 4th, 7th, 14th, and 21st days of the cultivation stage, culture medium from the AP and BL surfaces of the cells was respectively collected and transferred into 1.5 mL EP tubes to create cell samples. The ALP activity was then determined following the steps outlined in the ALP bioactivity rapid detection kit (P0321M).

#### 2.3.5 Permeability determination of Caco‐2 cell monolayer model

Phenol red is a water‐soluble substance with a relatively large molecular weight. Therefore, its transport through cell channels is challenging. It can be used as a marker to detect the permeation quantity of substances in the cell monolayer model, indirectly assessing the structural integrity of the cell monolayer model. Papp value reflects the ability of substances to pass through the monolayer cell barrier and the extent of their absorption.^12^ Therefore, experimental evaluators used phenol red solely as a marker in this identification process.

#### 2.3.6 Transwell drug transport experiment

PK levels were determined using double‐antibody sandwich ELISA, during which the ELISA kit instructions were referred.

The concentration of the standard sample (μg/mL) was used as the *x*‐axis and the corresponding optical density (OD) values as the *y*‐axis. A linear regression curve was established based on the standard sample. The sample concentration was then calculated based on this curve, allowing for the subsequent calculation of the cumulative permeation *Q* per unit area. The formula is as follows:

(3) $$Q_{n}=\frac{{\mathrm{VC}}_{n}+\sum_{i=1}^{n-1}C_{i}V_{i}}{A}$$

Among them, *A* represents the transport membrane area (0.33 cm ^2^), *V* denotes the volume of the Transwell inserts on the BL side, *C*_n_ signifies the drug concentration on the BL side during the n^th^ sampling, *C*_i_ represents the drug concentration on the BL side during the i^th^ sampling, and *V*_i_ stands for the volume during the i^th^ sampling.

##### Solution preparation

Ten milligrams of SNAC and 10 mg of PK were precisely weighed and diluted using PBS solution to achieve the desired concentration. The concentration of SNAC was 1000 μg/mL. The test group used SNAC with concentrations of SNAC and PK at ratios of 32:1, 16:1, 8:1, 4:1, and 2:1, respectively. while the control group did not contain SNAC.

After successfully establishing the Caco‐2 cell monolayer transport model, the culture medium from the upper and lower portions of the Transwell inserts was gently suctioned and removed. Subsequently, the upper and lower portions of the Transwell inserts were rinsed twice with preheated PBS solution at 37°C. Following rinsing, 350 μL of PBS solution was added to the AP side, while 1 mL of PBS was added to the BL side. The Transwell inserts were then incubated for 30 min in a cell culture incubator. Following incubation, the PBS solution from the AP and BL sides of the inserts were discarded. For both the test and control groups, 350 μL of the sample solution was added to the AP side of the Transwell inserts, while 1 mL of PBS solution was added to the BL side. For the blank group, a corresponding volume of PBS solution was added to both the AP and BL sides. Subsequently, the inserts were returned to the cell culture incubator, and samples were collected at 30, 60, 90, and 120 min. Each sampling instance involved withdrawing 200 μL of the mixed solution and replacing it with an equal volume of PBS. At 120‐min sampling, both the upper and lower portions of the Transwell inserts were fully collected, then transferred to centrifuge tubes, and stored in a −20°C freezer for subsequent processing.

##### PK standard curve

Linear regression was performed using PK concentration (*x*) and OD values (*y*) to plot the standard curve.

##### Transport experiment

Using a PK drug solution without SNAC as a control, drug transport experiments were conducted using a PK drug solution containing SNAC in the Caco‐2 cell model for 2 h. Samples were collected at 30, 60, 90, and 120 min, followed by the use of an ELISA assay kit to detect and measure the PK concentration in the samples. The cumulative permeation quantity (Q) and the cumulative permeation rate (%) per unit area were calculated.

### 2.4 Pharmacokinetic study of PK enteric‐coated capsules

#### 2.4.1 Processing of plasma samples

The experimental animals were divided into blank group, control group, and experimental group, with six rats in each group, and all of them were given a PK enteric‐coated capsule with a titer of 15 U.

Blood from the rat's retro‐orbital venous plexus at 0.3 mL was collected using a 0.5 mm capillary tube and transferred into a centrifuge tube that had been infiltrated and rinsed using 0.6 mL heparin sodium injection solution and dried. After being placed for 20 min, the blood sample underwent centrifugation at 1500 g for 20 min. Following centrifugation, a pipette was utilized to extract the supernatant, and the resulting plasma was stored in a freezer set at −20°C for subsequent use.

#### 2.4.2 Preparation and assessment of PK enteric‐coated capsules

According to the recommended daily dosage of PK enteric‐coated tablets for adults, the equivalent dosage for rat administration is 15 U/each, signifying that each enteric‐coated capsule contains 0.17 mg of PK.

The control group without SNAC: 9.83 g of starch were precisely weighed and placed in an agate mortar. Meticulously weighed PK, totaling 0.17 g, was added to the starch. The mixture was ground slowly for 1.5 h. After confirming the uniformity of the mixture, it was filled into empty capsules and then subjected to enteric coating.

The test group with SNAC (SNAC׃ PK = 32:1): 4.39 g of starch were precisely weighed and placed in an agate mortar. Subsequently, 5.44 g of SNAC were added to the agate mortar, followed by the precise addition of 0.17 g of PK. The components were thoroughly mixed together and ground slowly until achieving uniformity. Upon confirming the uniformity of the mixture, it was filled into empty capsules and then underwent an enteric coating process.

The upper, lower, left, right, and middle portions of the mixed powder were gathered and recombined. It was deemed to meet the standard if the RSD was less than 10%.

#### 2.4.3 Determination of PK concentration in plasma using the ELISA method

The ELISA kit instruction manual was referred.

#### 2.4.4 Methodological evaluation

##### Preparation of standard solution

Ten milligrams of PK were accurately weighed, placed in a 10 mL volumetric flask, dissolved in PBS solution, made up to the mark, and prepared into a standard solution with a concentration of 1 mg/mL.

##### Assessment of specificity

The PK standard solution with a concentration of 1 mg/mL was diluted by a specific factor to produce standard solutions used for preparing plasma at concentrations of 1200, 600, 300, 150, and 75 ng/L. The PK standard solution, with a concentration of 1 mg/mL, was diluted using blank rat plasma to create plasma samples that contained standard solutions and had concentrations of 1200, 600, 300, 150, and 75 ng/L. The OD values of the PK standard solution, plasma samples containing standard solutions, and blank plasma were measured using the ELISA method.

##### Investigation of linearity range

Plasma samples that contained standard solutions and had concentrations of 1200, 600, 300, 150, and 75 ng/L were prepared. The OD values of PK were measured using the ELISA method. Linear fitting was performed using PK mass concentration as the y‐axis and OD values as the *x*‐axis. The linearity within the range of 75–1200 ng/L met the experimental requirements.

##### Investigation of recovery rate

Plasma samples that contained standard solutions and had concentrations of 1000, 500, and 100 ng/L were prepared. Three replicates for each concentration were prepared. The OD values of PK were measured using the ELISA method. The recovery rate was measured as the measured value/added amount × 100%. An RSD <5% indicated good accuracy for this detection method.

##### Precision test

Plasma samples that contained standard solutions and had concentrations of 1000, 500, and 100 ng/L were prepared. Three replicates for each concentration were prepared. The OD values of PK were measured using the ELISA method for three consecutive days. Within‐batch and inter‐batch precision were assessed. An RSD of <10% was considered indicative of good precision.

##### Assessment of stability

Plasma samples that contained standard solutions and had concentrations of 1200, 600, and 150 ng/L were prepared. Three replicates for each concentration were prepared. The plasma samples were store at −20°C, and slowly thawed in a refrigerator at 4°C. The OD values of PK on the first, third, and seventh days were measured using the ELISA method. An RSD of <10% was considered indicative of good stability.

#### 2.4.5 Pharmacokinetic experiments

Rats were selected as experimental animals. The rats were divided into three groups: the blank control group, the group receiving SNAC‐containing PK enteric‐coated capsules, and the group receiving PK enteric‐coated capsules without SNAC, with six rats in each group. The rats fasted but were not water‐deprived 24 h before the experiment, as well as during the experiment. The three groups were orally administered. At 0.25, 0.5, 1, 2, 4, 6, 8, 10, and 12 h after administration, 0.3 mL of blood was collected from the rat retro‐orbital venous plexus using a 0.5 mm capillary tube. The collected blood was stored in a 0.6 mL EP tube that had been infiltrated with sodium heparin injection solution, and gently rocked up and down to prevent coagulation. The blood plasma was separated according to the method under section “2.4.1,” and stored at −20°C. The OD value of PK was determined using the ELISA method and substituted into the regression equation to obtain PK concentration in blood. Drug concentration–time curves were drawn using DAS 3.0 software.

### 2.5 Statistical methods

Statistical analysis, data processing, and computer‐generated plots were conducted using statistical analysis software such as Microsoft Excel 2010 and SPSS Statistics 21. The statistical analysis method—mean ± standard deviation ($\bar{x}\pm \mathrm{SD}$)—was directly employed to model and describe the differential data results statistically. Various differential data results obtained from single‐factor statistical analyses were synthesized and compared. Microsoft Excel 2010 was utilized for linear fitting to derive regression equations and plot standard curves. The software DAS 3.0 was used for pharmacokinetic analysis.

## 3 RESULTS

### 3.1 Comparison of the quality standards of synthesized SNAC

**FIGURE 2:**
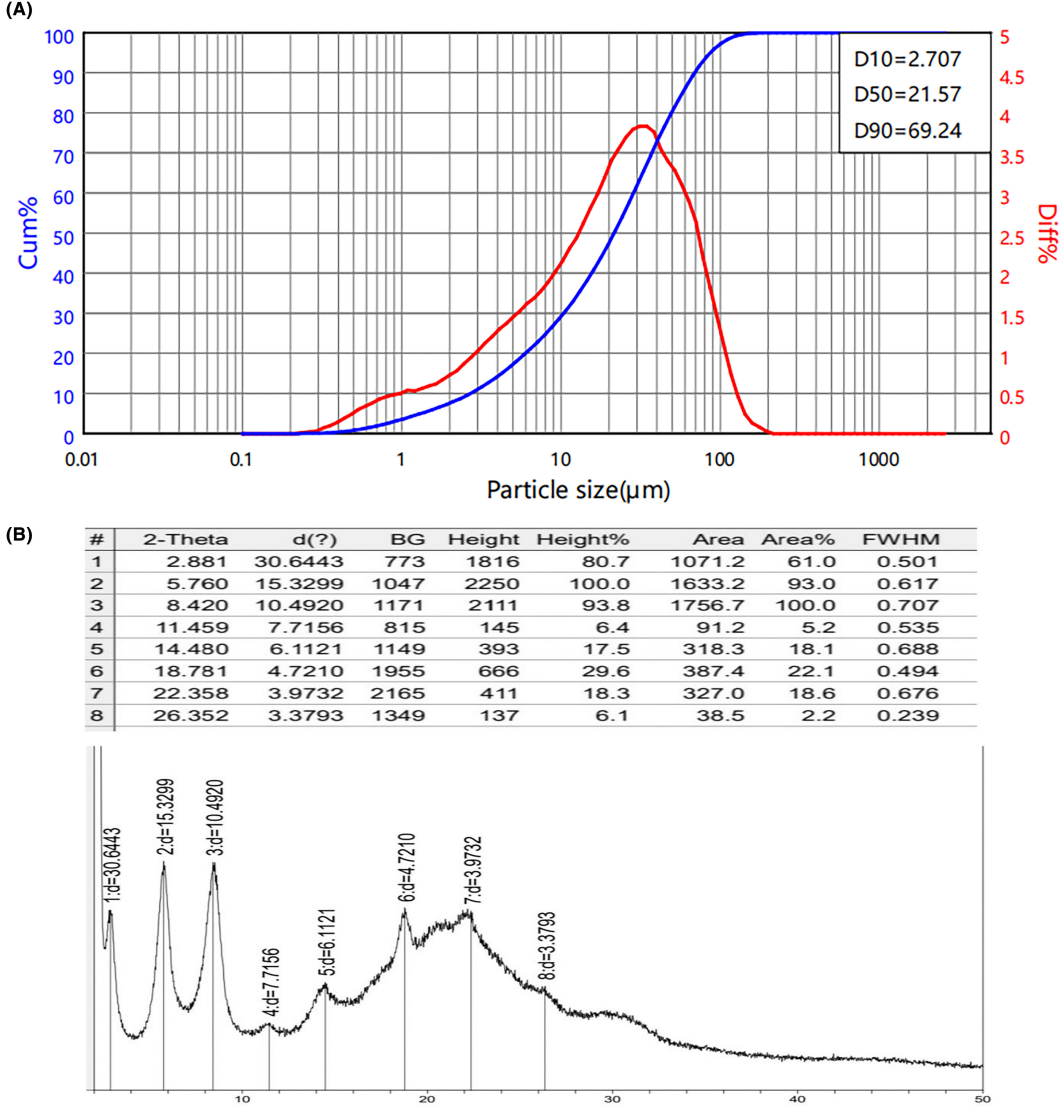
Granticle distribution plot (A) and crystalline form data (B) of SNAC.

As depicted in Table 1, all the properties and components of the synthesized SNAC met the respective quality standards, including characteristics, relevant substances, high‐resolution nuclear magnetic resonance (HNMR), moisture, and other parameters. Particle size distribution and crystallographic data are shown in Figure 2.

**TABLE 1:** Comparison of quality standards.

Item	Standard	Synthesized SNAC
Characteristics	Off‐white to pink powder	Off‐white powder
Relevant substance	Main peak ≥98.5%	Main peak = 99.9%
	Maximum individual impurity <0.1%	Maximum individual impurity = 0.02%
HNMR	No apparent impurities	Conforming
Moisture	Less than 0.2%	0.06%
Heavy metals	Not exceeding 20/1 000 000	Conforming
Residual solvents	Methanol ≤0.3%	Methanol = 0.01%
	Isopropanol ≤1.0%	Isopropanol = 0.48%
Particle size	D50 = 10 ~ 23 μm	D50 = 21.57 μm
XRD	In conformity with noncrystalline Type I	Conforming

### 3.2 SNAC promotes intestinal absorption of PK

#### 3.2.1 Results of cytotoxicity experiments

As indicated by the test results, when the concentration of SNAC reached 1000 μg/mL, the survival rate of Caco‐2 cells was higher than 95%. Therefore, within the range of 0–1000 μg/mL, the toxicity of SNAC to Caco‐2 cells within 6 h is very weak and can be considered negligible. When the concentration of PK reached 1200 μg/mL, the survival rate of Caco‐2 cells was higher than 100%. Consequently, within the range of 0–1200 μg/mL, PK exhibits no toxicity within 6 h.

#### 3.2.2 Caco‐2 cell model

**FIGURE 3:**
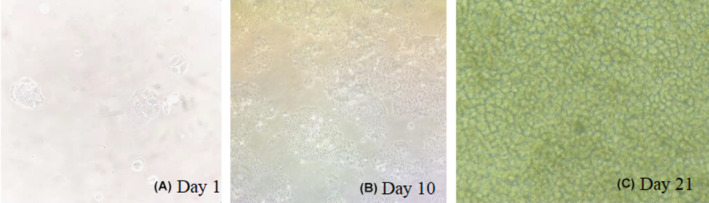
Morphological structure diagram of Caco‐2 cells. (A) Day 1 (B) Day 10, and (C) Day 21.

Caco‐2 cell morphology on Transwell culture dishes was observed using an inverted microscope and the results are displayed in Figure 3. The cell growth rate was relatively uniform with very distinct boundaries. Good intercellular connections were present. As the cells continued to proliferate, covering the walls of the culture dish, they exhibited a cobblestone‐like appearance, which can be utilized for subsequent experiments.

#### 3.2.3 Caco‐2 cell monolayer TEER

With the increase in days of cell culture, TEER gradually increased. By the 21st day, TEER reached 976.5 Ω*cm^2^. Moreover, the resistance remained stable before and after the transport experiment, demonstrating that the Caco‐2 cell monolayer model constructed in this experiment was tight and exhibited a good level of cell integrity.

#### 3.2.4 The ALP activity

With increases in cell culture duration, a notable increase in the ratio of ALP activity was observed between the cells on the AP side and those on the BL side. This result demonstrates the expression of APK on the AP side and consequently confirms the occurrence of polarization. A ratio of AP to BP greater than 3 indicates the completion of polarization and differentiation of Caco‐2 cells. (Table 2).

**TABLE 2:** Relationship of ALP activity on the AP and BL sides of the monolayer of Caco‐2 cells to culture time ($\bar{x}\pm \mathrm{SD}$, *n* = 3).

Time (days)	AP	BP	AP/BP
4	2.938 ± 0.129	2.078 ± 0.185	1.425 ± 0.198
7	6.984 ± 0.442	3.693 ± 0.553	1.911 ± 0.228
14	8.073 ± 0.485	2.119 ± 0.184	3.816 ± 0.122
21	11.460 ± 2.189	1.813 ± 0.284	6.305 ± 0.381

#### 3.2.5 Permeability of the Caco‐2 cell monolayer model

The permeation was determined at day 21 of the experiment, which was a more accurate determination that confirmed the integrity of the Caco‐2 cell model, as depicted in Table 3.

**TABLE 3:** Results of permeation using phenol red ($\bar{x}\pm \mathrm{SD}$, *n* = 3).

Items	Results
Regression equation	*y* = 0.0084*x* + 0.0512
Correlation coefficient (*r*)	0.9996
Papp(cm/s)	7.06 × 10^−8^
Reference value	<1 × 10^−6^

#### 3.2.6 Results of Transwell drug transport experiment

PK standard curve: A linear regression was performed using PK concentration (*x*‐axis) and OD value (*y*‐axis). The equation of the standard curve is *y* = 0.0208*x* + 0.168 (*r* = 0.9995). A concentration range of 7.5–120 ng/mL showed good linearity.

**FIGURE 4:**
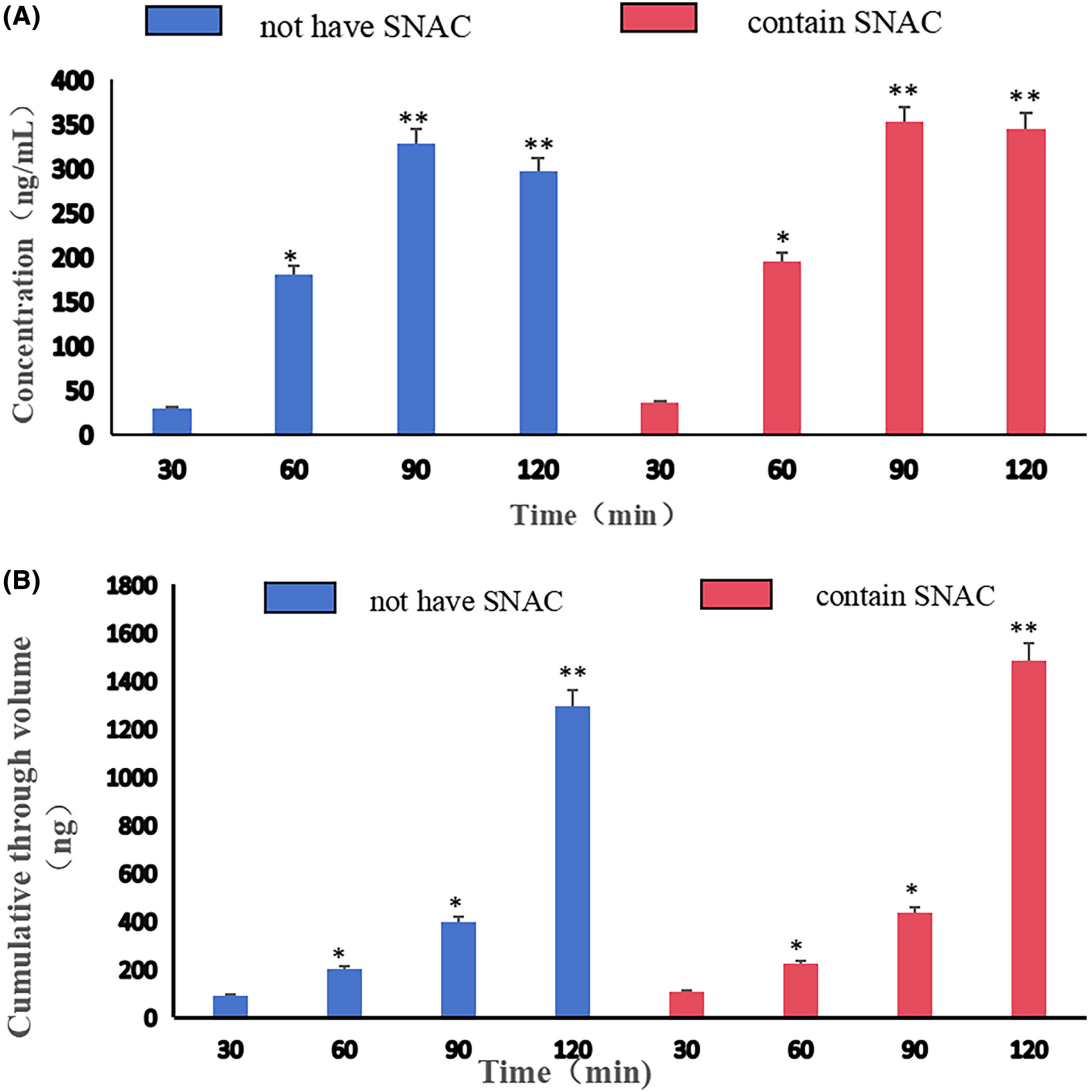
Concentration of PK (A) and cumulative permeation (B) at different time points (SNAC:PK = 32:1) ($\bar{x}\pm \mathrm{SD}$, *n* = 3). *P<0.05 when compared with the group that did not use SNAC at 30 min.** *P*<0.01 when compared with the group that did not use SNAC at 30 min. *P < 0.05, **P<0.01.

Based on the transport results, the test groups that used a SNAC to PK ratio of 32:1, 16:1, 8:1, 4:1, and 2:1 experienced a significantly higher cumulative PK permeation quantity than the control group without SNAC, with the cumulative PK permeation quantity increasing by 14.50%, 9.50%, 26.8%, 5.27%, and 3.9%, respectively. SNAC demonstrated an enhancing effect on the transport of PK, and the most effective ratio was SNAC:PK = 8:1. This result is similar to the findings in the research of Buckley S T^13^ et al. studying the interaction between SNAC and GLP‐1 drugs. However, the permeation rate was low. The highest cumulative permeation rate was observed at SNAC:PK = 1:1 (Figure 4).

### 3.3 Pharmacokinetics of PK enteric‐coated capsules

#### 3.3.1 Evaluation of the preparation results of PK enteric‐coated capsules

PK potency averages, with no SANC and SNAC at a ratio of 32:1, were 1.58 and 0.87 U/mg, respectively. The RSDs were 5.31% and 3.04%, respectively, both less than 10%, meeting the requirements.

#### 3.3.2 Methodological evaluation

##### Assessment of specificity

The results indicated that the OD values of the PK standard solution were similar to the plasma containing standard solutions. The OD values of the blank plasma were similar to those of the control blank wells, suggesting that neither the blank plasma nor the blank wells interfered with the PK determination. The results are displayed in Table 4.

**TABLE 4:** Detection results of various solutions using ELISA (OD value).

PK mass concentration (ng/L)	Standard solution	Plasma containing standard solutions	Blank plasma	Blank wells
1200	1.764	1.516	0.041	0.041
600	0.798	0.713	0.040	0.040
300	0.427	0.309	0.041	0.039
150	0.231	0.207	0.042	0.042
75	0.120	0.101	0.041	0.041

##### Assessment of linearity

Linear regression was performed using PK mass concentration as the *y*‐axis and OD values as the *x*‐axis to obtain the regression equation—*y* = 790.53*x* + 14.712 (*r* = 0.9978). The results indicated that the linear range of mass concentration for PK detection was 75–1200 ng/L, meeting the experimental requirements.

##### Assessment of recovery rate

As shown by the test results, the RSD of plasma samples that contained standard solutions and had concentrations of 1000, 500, and 100 ng/L were 0.561%, 0.813%, and 2.354%, respectively (*n* = 3). All of them were less than 5%, meeting the experimental requirements.

##### Precision test

As demonstrated by the test results, the within‐batch RSD of plasma samples that contained standard solutions and had concentrations of 1000, 500, and 100 ng/L were 5.01%, 3.04%, and 3.77%, respectively (*n* = 3), while those of the inter‐batch RSD were 4.39%, 3.47%, and 4.19%, respectively (*n* = 9). All RSDs were less than 10%, meeting the experimental requirements.

##### Assessment of stability

As indicated by the results, the RSDs of OD values of plasma samples that contained standard solutions and had concentrations of 1200, 600, and 150 ng/L were 3.14%, 7.36%, 8.24%, respectively (*n* = 9). All of them were less than 10%, proving good stability of PK under these conditions.

#### 3.3.3 Pharmacokinetics

**FIGURE 5:**
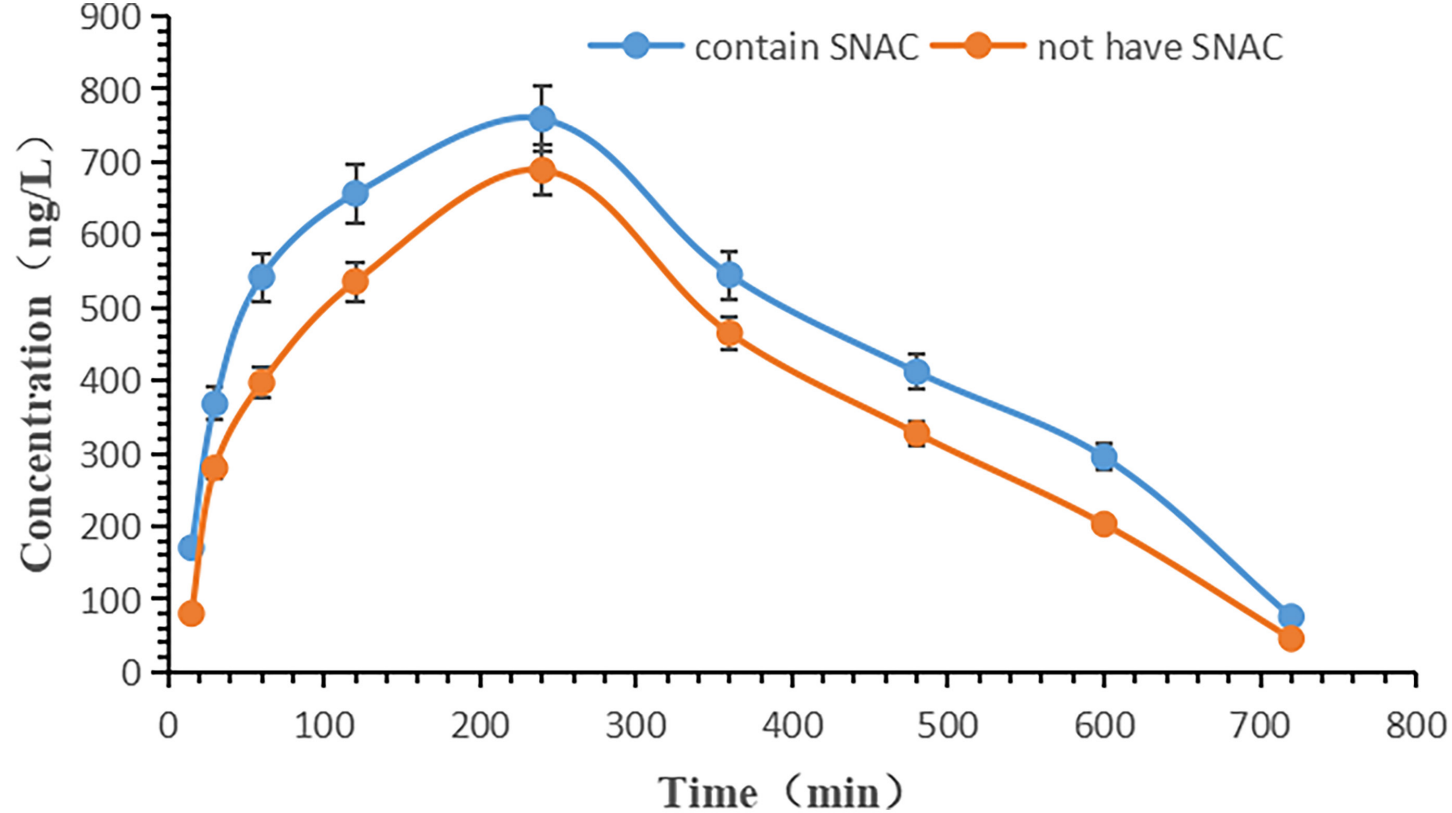
The PK concentration–time curve of rat blood in vivo.

As shown by the test results (Figure 5), in the rats' bodies, the PK in the PK enteric‐coated capsules that contained SANC had a pharmacokinetic AUC_0‐12 h_ of 5679.747 ng/L*h and a pharmacokinetic *t*_1/2_ of 4.569 h, while the PK in the PK enteric‐coated capsules that did not contain SANC had a pharmacokinetic AUC_0–12 h_ of 4639.665 ng/L*h and a pharmacokinetic *t*_1/2_ of 3.13 h. This indicates that the addition of SANC increased the AUC by 22%. Moreover, the *C*_max_ in the group using PK enteric‐coated capsules that contained SANC was higher than in the group using PK enteric‐coated capsules that did not contain SANC. This indicated that the inclusion of SANC, a permeation enhancer, allows more active ingredients to reach the site of action, thereby exerting efficacy. This suggests that the use of SANC can increase the PK concentration in the rat blood, offering advantages over formulations lacking SANC. This lays the groundwork for the future development of PK as a new type of orally administered second‐class drug.

## 4 DISCUSSION

ELISA is a type of microanalysis technique that combines the amplifying effect of enzyme catalysis with the specific antigen–antibody reaction. It is a commonly employed method that integrates high‐throughput screening with quantitative detection, suitable for analyzing large batches of samples. Its applications are widespread, encompassing fields such as immunological experimental analysis, medical diagnostics, and food safety.^14^ The principle of ELISA relies on the specific interaction between antigens and antibodies, utilizing enzyme labeling and chromogenic reactions. It utilizes immune methods for both qualitative and quantitative determinations, demonstrating high sensitivity capable of detecting levels as high as nanograms (ng) or even picograms (pg). Its advantages include strong specificity, high accuracy, straightforward operation, and safety.^15^, ^16^, ^17^ However, ELISA requires strict control of time and temperature, which can lead to poor reproducibility and susceptibility to interference from endogenous antibodies and enzymes. Therefore, in this study, methodological investigations were conducted regarding the linear range, recovery rate, accuracy, and precision of ELISA quantitative measurements, all of which met the standard requirements.

The absorption enhancer selected in this study was SNAC, primarily used to overcome the absorption barriers of drugs and enhance the bioavailability of specific orally administered protein and peptide (or polypeptide) drugs. SNAC, as an amino acid derivative, can increase the oral absorption of various protein drugs such as heparin and human growth hormone without relying on drug formula protection. Moreover, it does not have a short‐term impact on the vitality of gastrointestinal cells.^18^, ^19^ This research on SNAC is mainly limited to protein drugs, with most studies investigating its absorption‐enhancing effects in the stomach. This study focused on the effect of SNAC on enhancing the absorption of PK, providing a theoretical basis for its role in promoting drug absorption in the intestine and investigating the impact of SNAC on the vitality of intestinal cells. SNAC possesses enormous potential for future applications. As a broad‐spectrum absorption enhancer, it can be used not only to promote drug absorption and develop new types of drugs but also holds potential applications in other areas.

The most important aspect of the research on the Caco‐2 cell absorption model is the establishment of a monolayer membrane. Therefore, evaluating the integrity and tightness of the membrane is crucial. There are various evaluation methods. Besides the ALP method and the phenol red method mentioned in this article, there are other evaluation methods such as cell resistance determination, mannitol permeation, electron microscopy for ultrastructure observation, etc. Overall, during the modeling process, two or more methods are needed, with the goal of reducing errors and providing a more comprehensive assessment of the monolayer membrane's tightness and integrity. The Caco‐2 cell absorption model method holds substantial potential applications, progressively assuming a pivotal role in advancing drug development. It can also be applied to other areas, such as exploring the bioavailability of microplastics and food development. Some studies have found the presence of microplastics in the human body,^20^ with the majority being absorbed through the intestines into the bloodstream. The inverted model can be used for validation and further exploration of this phenomenon.

## 5 CONCLUSION

This study has successfully designed and optimized SNAC synthesis route that generates intermediate products with reduced production costs while ensuring safety, environmental sustainability, ease of experimental operation, and facilitation of purification processes, rendering it amenable for industrial‐scale production. Additionally, it validates the absorption‐enhancing effect of SNAC on PK, thereby enhancing its bioavailability within the body. This work lays a theoretical foundation for the development and subsequent research of second‐class new drugs. At present, only male rats are conducted, and the study of gender differences can be continued in the future.

## AUTHOR CONTRIBUTIONS

All authors made substantial contributions to the design of this study, data acquisition and interpretation, statistical planning, drafting the article, or revising the article critically. All authors agree to be accountable for all aspects of the work and have approved the final version of the article for submission.

## FUNDING INFORMATION

None.

## CONFLICT OF INTEREST STATEMENT

The authors declare that they have no conflicts of interest.

## ETHICS STATEMENT

Not applicable.

## Data Availability

The datasets used and/or analyzed during this study are available from the corresponding author on reasonable request.
